# Fructan Biosynthetic and Breakdown Enzymes in Dicots Evolved From Different Invertases. Expression of Fructan Genes Throughout Chicory Development

**DOI:** 10.1100/tsw.2002.288

**Published:** 2002-05-11

**Authors:** Wim Van den Ende, An Michiels, Joke De Roover, Andrea Van Laere

**Affiliations:** Department of Biology, Laboratory for Developmental Biology, Botany Institute, KULeuven, Kasteelpark Arenberg 31, B-3001 Leuven, Belgium

**Keywords:** 1-FEH, 1-FFT, 1-SST, Chicory (*Cichorium intybus* L.), fructan, fructosyltransferase, inulin, invertase

## Abstract

Fructans are fructose-based oligo- and polymers that serve as reserve carbohydrates in many plant species. The biochemistry of fructan biosynthesis in dicots has been resolved, and the respective cDNAs have been cloned. Recent progress has now succeeded in elucidating the biochemistry and molecular biology of fructan biodegradation in chicory, an economically important species used for commercial inulin extraction. Unlike fructan biosynthetic genes that originated from vacuolar-type invertase, fructan exohydrolases (FEHs) seem to have evolved from a cell-wall invertase ancestor gene that later obtained a low iso-electric point and a vacuolar targeting signal. Expression analysis reveals that fructan enzymes are controlled mainly at the transcriptional level. Using chicory as a model system, northern analysis was consistent with enzymatic activity measurements and observed carbohydrate changes throughout its development.

### INTRODUCTION

Fructans occur as a reserve carbohydrate in about 15% of flowering plant species[1]. Fructans are defined as oligo- or polymeric carbohydrates in which fructosyl linkages predominate[2]. They are built up by further adding fructofuranosyl units (β(2→1) or β(2→6) linkages) to three basic trisaccharides (1-kestose, 6-kestose, and neokestose), which themselves are synthesized by linking a fructose moiety to one of the three primary hydroxyl groups of sucrose[3]. Dicotyledonous species store inulin-type fructan consisting of linear β(2→1) linked fructofuranosyl units. The most extensively studied species include Jerusalem artichoke (*Helianthus tuberosus*) and chicory (*Cichorium intybus*), which belong to the Asteraceae. However, inulin-type fructans also occur in some other dicot families (Boraginaceae, Campanulaceae). Levan-type fructan consists of linear β(2→6) linked fructofuranosyl units. More complex and branched fructan types, including graminan or mixed-type fructan as well as neokestose-based fructans, are common in monocotyledonous species[4]. Fructans are used for food and non-food applications[5]. So far, chicory is the only crop that is used for commercial fructan extraction. During the last decade, annual inulin production from chicory roots increased from 1,000 to more than 100,000 tons. Therefore, this review mainly focuses on inulin-type fructans and on inulin metabolism in chicory roots.

Besides their function as a reserve carbohydrate, fructans might fulfill other, perhaps more specific, roles in plants. Some data suggest a correlation with drought resistance, frost tolerance, and osmoregulation[1,6,7]. The molecular mechanism explaining the protective function of fructans is still obscure. However, it has recently been demonstrated that fructans stabilize liposomes during freeze- or air-drying[8,9]. Unlike starch, fructans are water soluble and are believed to be localized in the vacuole[10]. However, the exclusive vacuolar localization of fructan metabolism has recently been challenged, because fructans have been detected in the apoplast and even within xylem vessels[7,11,12].

### PLANT FRUCTAN BIOSYNTHESIS

#### Inulin Biosynthesis

Inulin-type fructans can be further divided into two classes: the regular inulin type (also termed GFn type) contains a terminal glucose moiety and the inulo-*n*-ose type (Fn type) lacks the terminal glucose moiety. GFn-type inulins are nonreducing and Fn-type fructans are reducing compounds.

In 1968, Edelman and Jefford proposed a model for the biosynthesis of GFn-type inulin in *H. tuberosus*[44]. The model claims that no phosphorylated precursors are involved in inulin biosynthesis and that sucrose is the only substrate for inulin synthesis in a process involving two distinct enzymes, which are termed fructosyltransferases (Fig. 1). Sucrose:sucrose 1-fructosyltransferase (1-SST; EC 2.4.1.99) transfers a fructose moiety from a sucrose to another sucrose molecule yielding the trisaccharide 1-kestose (GF + GF → GFF + G) in an essentially irreversible reaction. Further chain elongation is accomplished by the enzyme fructan:fructan 1-fructosyltransferase (1-FFT; EC 2.4.1.100), which is capable of transferring fructose moieties from 1-kestose (or larger fructans) to sucrose or other fructans ($GF_n + GF_m \leftrightarrow GF_{(n-1)} + GF_{(m+1)}$ with $n \geq 2$, $m \geq 1$). This reaction is reversible. Preferred donor and acceptor substrates for 1-FFTs might differ between plant species and could result in different inulin profiles (e.g., *Cynara* vs. *Helianthus*)[13,14].

The 1-SST/ 1-FFT model explains only the production of nonreducing inulins from sucrose. The origin of the reducing Fn-type fructans was obscure for a long time. It was believed that these inulo-*n*-oses resulted from a degradation process by endo-type plant fructan hydrolases or even from α-glucosidase activity. Extensive search has yielded no evidence of the existence of such enzymes in plants. However, using chicory as a model plant, it was demonstrated that inulo-*n*-

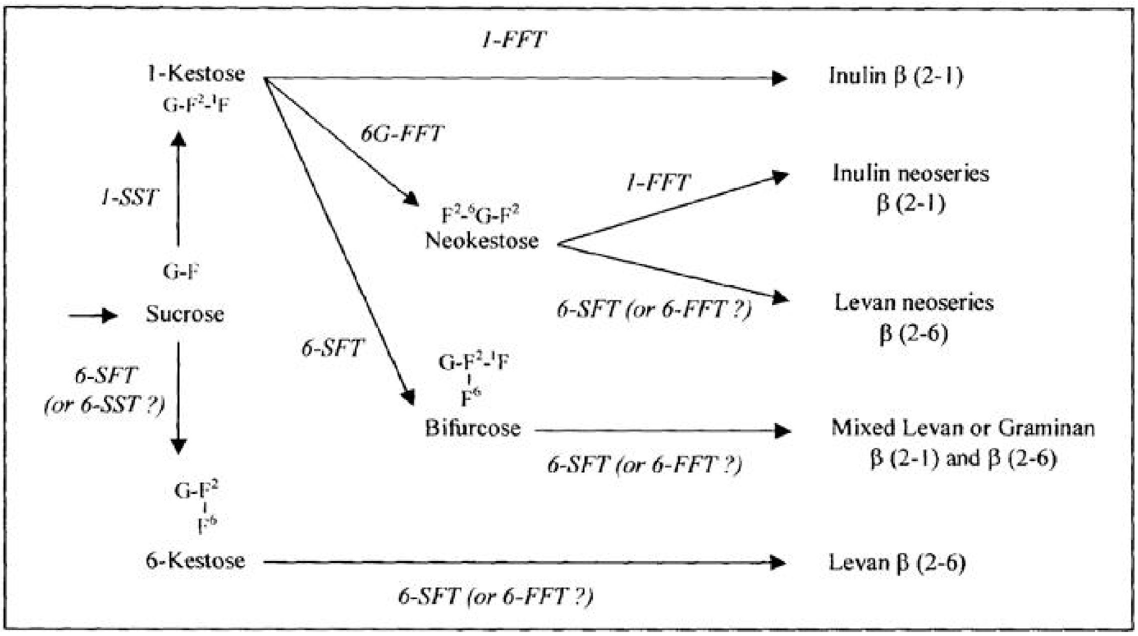

**FIGURE 1.** Schematic model of the enzymology of fructan biosynthesis in plants. The enzymes 6-FFT and 6-SST are between brackets, since their existence is not proved.

oses are not breakdown products, but on the contrary these molecules are biosynthesized by 1-FFT using free fructose (produced by 1-FEH activity, see below) or Fn-type fructans as acceptor substrates[15]. This means that 1-FFT can use either free fructose or Fn-type fructans next to GFn-type fructans. Therefore, the 1-FFT catalyzed reaction can now be extended as follows: $GF_n + (G)F_m \leftrightarrow GF_{(n-1)} + (G)F_{(m+1)}$ with $n \geq 2$, $m \geq 1$.

### Levan and Graminan Biosynthesis

For species that accumulate unbranched levan (e.g., *Dactylis glomerata*[16]), it is still uncertain whether the biosynthesis occurs by only a single enzyme, namely, a 6-SFT, or that specific 6-SST and 6-FFT enzymes, which have not yet been isolated, are involved (Fig. 1). Sprenger et al. first characterized 6-SFT from barley[17], a species that preferentially stores mixed-type levans. This enzyme uses sucrose only as a donor substrate and creates β(2→6) linkages. Since 1-kestose (and not sucrose) is the preferred fructosyl acceptor for 6-SFT[17], it became clear that the enzyme 1-SST fulfills an important role during mixed-type levan synthesis as well as during inulin biosynthesis (Fig. 1). In strong contrast to inulin biosynthesis, it was suggested that mixed-type levans can be "trimmed" by specific fructan hydrolases (1-FEH, 6-FEH, see below) that are coexpressed throughout the period of fructan biosynthesis[18].

### Synthesis of Neokestose-Based Fructans

Species belonging to the Liliaceae (e.g., *Allium cepa*, *Asparagus officinalis*) as well as some species belonging to the Poaceae (e.g., *Avena sativa, Lolium perenne*) contain fructans in which the glucose is internal in the chain[19,20,21]. For the biosynthesis of this kind of fructans, an additional fructosyltransferase is needed: $6^G$-FFT[22,23]. This enzyme is capable of transferring a fructose moiety to the C6 position of the glucose molecule in sucrose, in that way synthesizing the trisaccharide neokestose. Neokestose can be further elongated on either side of the internal glucose by the activity of 1-FFT (inulin neoseries) or 6-SFT (levan neoseries). For a good overview of the enzymology of graminan as well as neokestose-type fructans in grasses, we refer to recent work on *L. perenne*[21,24]. A schematic overview of the biosynthesis of the different types of fructans is presented in Fig. 1.

### Validity of the 1-SST/ 1-FFT Model in Asteraceae

Without any doubt, inulin is the most investigated fructan type. Although the 1-SST/1-FFT model for inulin biosynthesis has been a subject of criticism[25], the validity (or at least the feasibility) of the model has been demonstrated when it was shown with both chicory[26] and Jerusalem artichoke–purified 1-SST and 1-FFT enzymes[27,28] that it was possible to synthesize *in vivo*-type fructans *in vitro* starting from physiologically relevant sucrose concentrations. The problem remained that the degree of polymerization (DP) obtained *in vitro* was always lower than that found *in vivo*. However, this difference could be explained by sucrose depletion *in vitro*, the accumulation of glucose, and the instability of the enzymes after longer incubation periods. Further proof of the validity of the 1-SST/1-FFT model came from the heterologous expression of the cDNAs for 1-SST and 1-FFT into potato[14] or sugar beet[29]. At least in potatoes, it was convincingly demonstrated that the introduction of 1-SST and 1-FFT from *Cynara scolymus* resulted in the same fructan profiles as occurring in *C. scolymus*[14]. However, the results obtained are still a subject of criticism[30], since it was argued that the amounts of fructans as well as the enzymatic activities derived from transgenic plants are much lower than normally observed within fructan plants. Moreover, the same phenomenon is also observed when comparing *in vitro* and *in vivo* biosynthesis rates, the latter often being several times higher. A possible explanation for the observed difference could be the higher instability of the fructan biosynthetic enzymes in a “strange” environment (such as a glass tube or non-fructan plant). Fructan biosynthetic enzymes are believed to operate in the vacuole[9]. However, so far it cannot be excluded that fructan biosynthesis could occur in small, prevacuolar compartments[31] containing very high sucrose and/or enzyme concentrations and that such specific conditions cannot be reached in non-fructan plants.

### Cloning of Fructan Biosynthetic cDNAs

The first fructosyltransferase cDNA cloned was the 6-SFT from barley[17]. Later, 1-SST and 1-FFT were cloned from *Cichorium intybus*[32,33], *Cynara scolymus*[34,13], and *H. tuberosus*[35]. Over the same period, 1-SST and $6^G$-FFT cDNAs were obtained from *Allium cepa*[36,37]. Later, the 1-SST cDNAs from *Taraxacum officinale*[12] and *Festuca arundinacea*[38] were isolated. Recently, a great number of (partial) 6-SFT cDNAs were obtained from a variety of grass species[39]. Typically, fructan biosynthetic cDNAs, just like vacuolar invertases[40], contain a relatively long signal peptide that might contain an extra signal for vacuolar targeting[41,12]. Although, in general, sequences are more divergent in this signal peptide region compared to the mature protein region, a literature survey of all known vacuolar invertases and plant fructosyltransferases (also including a number of 5’ expressed sequence tags) revealed that all dicot cDNAs and most monocot cDNAs contain the conserved sequence [S/A/P]**R**[G/A/P]XXX**GVS**[D/E/M]**K**[S/T/A] in this region. Furthermore, all known vacuolar-type invertases (but also all monocot fructan biosynthetic enzymes) have the conserved sequence **W**XXX[M/I/V]**L**X**WQ** around the region where the mature protein starts, while extracellular invertases clearly lack this region[41].

### Fructan Biosynthetic Enzymes are Derived from Vacuolar Invertases

Fructan biosynthetic enzymes are more similar to vacuolar-type invertases (42 to 56% identity) than to cell-wall-type invertases (36 to 39% identity). Hence, fructan plants seem to have recruited their fructan biosynthetic genes from vacuolar invertases by relatively small mutational changes[42]. Three groups of vacuolar-type enzymes become apparent (Fig. 2A), suggesting repeated duplication of the ancestral gene. The third group contains only dicotyledonous invertases. The second group harbors only monocot enzymes. Surprisingly, part of the first group (Ia) also contains three monocot invertases from rice (AF019113, AY037870, and AY037871). Therefore, it can be hypothesized that this group of enzymes still resembles more the common ancestral vacuolar invertase that existed before monocots and dicots were separated. In this regard, dicot fructan biosynthetic enzymes (Ib) are clearly derived within this group, while monocot fructan biosynthetic enzymes (II) are not, suggesting that fructan biosynthetic enzymes evolved independently in monocots and dicots throughout evolution. For this reason, e.g., monocot 1-SST is more identical to the invertase from which it has evolved (e.g., *A. cepa* 1-SST and *A. cepa* invertase ACE6067; Fig. 2A) than to all dicot 1-SSTs. This phylogenetic analysis is consistent with the polyphyletic origin of the capacity for fructan synthesis in contemporary plant species[1] and the fact that different enzymes (6-SFT vs. 1-FFT) play a key role in fructan metabolism in monocots compared to dicots. All three vacuolar invertases found in the genome of *Arabidopsis thaliana* belong to group III, and, surprisingly, no representative of group I seems to be present within this species. Interestingly, one rice cDNA-derived sequence (AF276704) groups together with fructosyltransferases, although no fructans accumulate in rice. Therefore, it would be useful to fully investigate the functionality of this clone being an invertase or a fructosyltransferase.

## PLANT FRUCTAN BREAKDOWN

### FEH Enzymes

In plants, fructan breakdown into sucrose and fructose is accomplished by the action of fructan exohydrolases (FEH; $GF_n + H_2O \Rightarrow GF_{(n-1)} + F$ with $n > 1$). Unlike in bacteria, no fructan endohydrolases seem to exist in plants. Further breakdown of sucrose to hexoses might occur by the action of acid invertases ($GF \rightarrow G + F$; EC 3.2.1.26). In severe contrast to real FEHs, which are capable of degrading only fructans and are unable to degrade sucrose, β-fructo(furano)sidases (EC 3.2.1.80) can degrade sucrose as well as fructans, but their presence in plants is far from clear, since, at least in some cases, the obtained enzyme preparations could still have been a mixture of both invertase and fructan exohydrolase[43]. In our opinion, plant FEHs are clearly different from β-fructo(furano)sidases; therefore, they cannot be classified under EC 3.2.1.80.

Depending on the linkage type that is broken, one can discriminate 1-FEH ($\beta 2 \rightarrow 1$) from 6-FEH ($\beta 2 \rightarrow 6$) enzymes. Although the activity is well characterized in various plant species[43,44], only a few plant FEH enzymes have been purified to electrophoretic homogeneity. They include three fructan 1-exohydrolases from chicory (1-FEH I, IIa, and IIb)[45,46,47], a 1-FEH from *H. tuberosus*[48], a fructan 6-exohydrolase (6-FEH) from *L. perenne*[49], a FEH that preferentially hydrolyzes $\beta(2 \rightarrow 6)$ linkages (*Avena sativa*[50]), and another FEH that is able to attack both $\beta(2 \rightarrow 1)$ and $\beta(2 \rightarrow 6)$ linkages (*Hordeum vulgare*[51]). Chicory 1-FEH I differs only slightly from the 1-FEH II iso-enzymes in its kinetic properties and inhibition by sucrose[46].

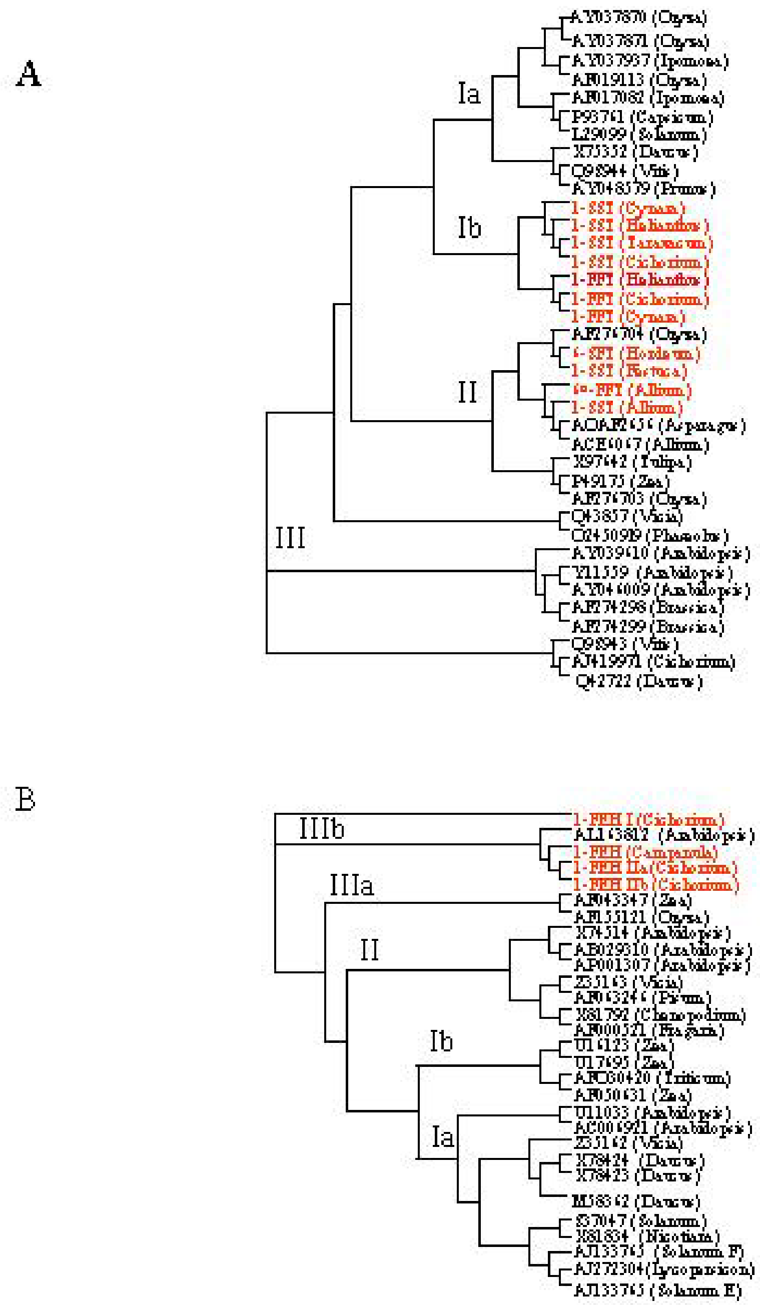

**FIGURE 2.** Rooted phylogenetic trees containing cDNA-derived amino acid sequences of plant vacuolar-type invertases and fructosyltransferases and cDNA-derived amino acid sequences of plant cell-wall–type invertases and FEHs. **Within the vacuolar enzymes (A)**, three groups can be discerned. First group (Ia): invertases of *Oryza sativa, Ipomoea batatas, Capsicum annuum, Solanum tuberosum, Vitis vinifera*, *Daucus carota,* and *Prunus cerasus.* First group (Ib): dicot fructan biosynthetic enzymes 1-SST and 1-FFT from *Helianthus tuberosus, Cichorium intybus,* and *Cynara scolymus* and 1-SST from *Taraxacum officinale*. Second group (II): monocot fructan biosynthetic enzymes and invertases from *Hordeum vulgare* (6-SFT), *Allium cepa* (1-SST, $6^G$-FFT and invertase), *Festuca arundinacea* (1-SST), *Asparagus officinalis, Zea mays*, *Tulipa gesneriana,* and *Oryza sativa.* Third group (III): all other dicot invertases of *Cichorium intybus*, *Daucus carota*, *Arabidopsis thaliana*, *Brassica oleracea, Vicia faba*, *Phaseolus aureus,* and *Vitis vinifera*. **Within the cell-wall invertases (B),** three groups can be differentiated. First group (Ib): monocot cell-wall invertases from *Zea mays* and *Triticum aestivum*. First group (Ia): dicot cell-wall invertases from *Vicia faba, Daucus carota, Solanum tuberosum, Lycopersicon esculentum, Arabidopsis thaliana,* and *Nicotiana tabacum*. Second group (II): dicot cell-wall invertases from *Arabidopsis thaliana, Vicia faba, Pisum sativum, Chenopodium rubrum,* and *Fragaria* × *ananassa*. Third group (IIIa): low pI cell-wall invertases from *Zea mays* and *Oryza sativa.* Third group (IIIb): low pI *Arabidopsis thaliana* fructosidase and 1-FEHs from *Cichorium intybus* (1-FEH I, IIa, and b) and *Campanula rapunculoides*. Plant fructan metabolic enzymes are indicated in red. All other cDNA-derived sequences are believed to represent invertases. Their accession number is presented.

### Cloning of Plant FEHs

Although numerous FEH cDNAs were obtained from microorganisms[52], so far only three plant FEHs were cloned, namely, chicory 1-FEH I[41] and chicory 1-FEH IIa and b[47]. The FEH functionality was confirmed by heterologous expression in potato and/or Q-TOF analyses on tryptic fragments of the purified proteins. Recently, a new FEH clone was obtained from *Campanula rapunculoides*, a representative of the Campanulaceae family (unpublished results).

### Plant Fructan Breakdown Enzymes are Derived from Cell-Wall Invertases

Surprisingly, the FEHs from chicory and *C. rapunculoides* are much more similar to cell-wall invertases (43 to 59% identical amino acids) than to vacuolar invertases (40 to 41% identical). This implies that at least in dicots, 1-FEHs have evolved from an ancestral cell-wall invertase gene[41,47]. Three subgroups of cell-wall invertases become apparent (Fig. 2B), suggesting a repeated duplication of the ancestral cell-wall invertase gene. One subgroup containing the chicory and campanula 1-FEHs as well as the two monocot representatives AF043347 and AF155121 (IIIa and b, Fig. 2B) can be discerned from the other groups by their acidic pIs and the presence of an extra C-terminal stretch containing a putative vacuolar targeting signal[41]. This suggests an evolutionary pressure toward the development of "cell-wall-like" vacuolar fructan exohydrolases and/or invertases. The reasons why chicory fructan 1-exohydrolases have evolved from a cell-wall-type ancestral invertase and not from a vacuolar invertase gene are far from clear. It can be speculated that cell-wall invertase genes might have been better candidates for the development of genuine 1-FEHs, being capable of using oligo- or polysaccharides instead of sucrose as fructosyl donors. In this regard, it has been demonstrated that native purified extracellular invertases from carrot show a much higher cleavage rate for raffinose than carrot vacuolar enzymes[53 and references therein]. Moreover, it was recently demonstrated that the different substrate specificities of vacuolar invertases (containing the conserved WECVD motif) and extracellular invertases (containing the conserved WECPD motif) are fully determined by a single amino acid substitution (P to V[54]). All members of group III (Fig. 2B) contain a P in this conserved motif. It can be questioned whether all these represent fructan exohydrolases or that also invertases or β-fructosidases (e.g., AL163812 of *Arabidopsis thaliana*) are present. The development of good heterologous expression systems (e.g., expression in *Pichia pastoris*[55]) will allow a rapid determination of functionalities and opens perspectives to perform structure-function studies by site-directed mutagenesis.

Up to now, efforts to localize the cell-wall-derived 1-FEH enzymes did not point out an apoplastic localization, but favor a vacuolar localization.

### Plant Fructan Enzymes: Different Functionalities Still Evolving?

Most enzymes are biological macromolecules that catalyze very specific reactions by acting on very particular substrates. In this respect, fructan metabolic enzymes are somewhat peculiar in that they have their preferential donor and acceptor substrates, but they often catalyze other fructosyl transfers—although at a much lower efficiency—depending on the type and substrate concentrations provided, the temperature, and ionic strength[56 and references therein]. For instance, at high temperatures and with low substrate concentrations, 1-SST and 1-FFT show some β-fructosidase activity (transfer of fructosyl moieties to water instead of sucrose or fructan), and 1-SST also shows some 1-FFT activity. Besides small inulins and water, compounds like ethylene glycol and glycerol can also serve as acceptor substrates for chicory 1-SST[26]. On the other hand, under very high sucrose concentrations, small fructans can be synthesized by yeast invertase enzymes[25]. Moreover, with purified chicory root 1-SST and 1-FFT, it was demonstrated that the maximal DP that can be obtained *in vitro* depends on the enzyme concentration used. Because the final fructan pattern obtained *in vitro* is a function of the (1-SST + 1-FFT)/sucrose ratio, it can be suggested that the latter ratio *in situ* could affect the highly variable tissue- or species-specific pattern of fructans produced *in vivo*[57].

The possibly different functionalities of each enzyme severely complicate fructan enzyme nomenclature. Also, it is not easy to distinguish discrete enzyme activities when measured in crude extracts containing varying amounts of protein. For all these reasons, some fructan researchers prefer to use the general term "fructosyltransferases" or "invertase-like" enzymes and refuse to accept the 1-SST/1-FFT terminology. It can be postulated that 1-SST evolved from a vacuolar invertase that became able to use sucrose as an acceptor substrate. Possibly, 1-FFT later evolved from 1-SST and became able to use oligo- and polyfructans as a donor substrate. On the contrary, plant FEHs seem to have evolved from cell-wall–type invertases that became able to use oligo- and polyfructans as a donor substrate. Maybe plant β-fructosidases that can use either sucrose or fructans as donor substrates can be considered a kind of evolutionary "intermediate" between invertases and FEHs, but this remains to be further investigated. Noticeably, chicory 1-FEH I and II are the most specific enzymes, since they can use fructan only as a donor substrate (no invertase activity) and water only as an acceptor substrate.

Structure-function research on plant fructosyltransferases is still in its infancy. For bacterial levansucrase, an enzyme that catalyzes microbial fructan biosynthesis, it was demonstrated that one single-point mutation changes a bacterial levansucrase into an invertase[58]. Although it has become clear which amino acids might play a role in the active site formation (D in NDPNG; E in WEC(V/P)D, and D in FRDP)[59,60], structure-function research is severely hampered by the fact that it is difficult to obtain large amounts of pure enzymes for crystallization and X-ray analysis.

### FRUCTAN METABOLISM IN CHICORY

#### Carbohydrates

The introduction of ion exchange HPLC with pulsed amperometric detection at the beginning of the 1990s[61] was a tremendous step forward in the study of the structural diversity of fructans in many species. In chicory roots, high DP fructans are synthesized during summer growth. However, in early October, important changes occur over a very short period[62]. These changes include: (1) a significant increase in fructose concentration, (2) an increase in the concentration of fructans with a low DP, (3) a decrease in fructans with a high DP, and (4) an appearance of alternative peaks (representing Fn-type fructans). All these changes are highly disadvantageous for industrial fructan production, especially for high DP fructan production. The induction of 1-FEH activity in chicory roots necessitates a rather early harvest (around the end of September) and quick processing. Moreover, the resulting high fructose concentrations together with high 1-FFT activity (see below) invariably lead to the production of reducing low DP fructans lacking a terminal glucose (the $F_n$ series) and favoring unwanted Maillard reactions with amino acids during processing.

Fructans have also been reported in chicory leaves, but leaf fructans are smaller and consist predominantly of DP 3-5 polymers with a maximum DP of 12[63].

### Activity Determinations

During field growth, the activity of 1-SST in roots decreased continuously to essentially disappear in October (Fig. 3A1). The 1-FFT activities, on the other hand, remained high and even increased slightly (Fig. 3A1). In the absence of 1-SST, 1-FFT will use any incoming sucrose as an acceptor substrate for fructosyl units from larger fructans, resulting in a reduced average DP and an increase in molar concentration of fructans (although not of fructan weight) throughout the month of September. The increase in molar concentration might contribute to cold resistance and/or more efficient inulin breakdown (by increased substrate concentration).

Minor fructan exohydrolase activity was detected throughout field growth, but in crude extracts, a part of it is likely to be due to the β-fructosidase activity of 1-FFT[64]. The genuine 1-FEH activity rapidly increases after the first half of October (Fig. 3A2). During cold storage, a further rapid increase of 1-FEH activity was detected[64].

### Northern Analyses

To our knowledge, chicory is the only fructan species from which fructan biosynthetic (1-SST, 1-FFT) and fructan breakdown (1-FEH I, IIa, and IIb) cDNAs are cloned. This provides an excellent tool to study the expression of the genes throughout plant development. The 1-SST is highly expressed in young roots, but its expression decreases throughout the growing season, while 1-FFT appears to be constitutively expressed over the same period (Fig. 3B). In October, 1-FEH I and 1-FEH IIa expression are induced and expression levels increase after harvesting and cold storage.

For all enzymes, Northern blot profiles are consistent with enzymatic activity measurements (Fig. 3A) and carbohydrate profiles. Therefore, it can be suggested that all these chicory fructan enzymes are regulated mainly at the transcriptional level. The same conclusion could be drawn from a 1-SST expression analysis in different *Taraxacum* tissues[12] and from a 6-SFT expression analysis in barley[65].

### REGULATION OF FRUCTAN METABOLISM

As stated above, plant fructan genes as well as invertase genes[40] seem to be regulated mainly at the transcriptional level, although an additional post-translational regulation cannot be excluded (monomeric vs. heterodimeric enzyme ratios change during plant development)[66,53,12]. The endogenous and/or exogenous effectors responsible for gene induction are still the subject of investigation in many laboratories. So far, only the barley 6-SFT promoter has been published[67]. The exact regulatory pathways that control fructan metabolism are essentially unknown. However, it can be expected—like for plant acid invertases[68,69]—that the genes will be regulated developmentally by sugars, wounding, hormones, and stress factors (e.g., drought or low temperatures).

Previous studies on grass species[70,71,72] suggested that fructan biosynthesis and degradation depend upon the sucrose level in the cells. Experiments on roots of young chicory seedlings under N-stress as well as after defoliation revealed that fructan biosynthesis and breakdown are intimately connected with the sucrose levels in the root[73,74]. Fructan biosynthetic enzymes become operative in the sink root when sucrose is exported to the roots. This happens when the photosynthetic capacity of the leaves becomes higher than what is needed for leaf growth and maintenance. De Roover et al.[75] demonstrated that 1-SST activity and fructan biosynthesis are initiated when growth of chicory seedlings is arrested by drought. Direct

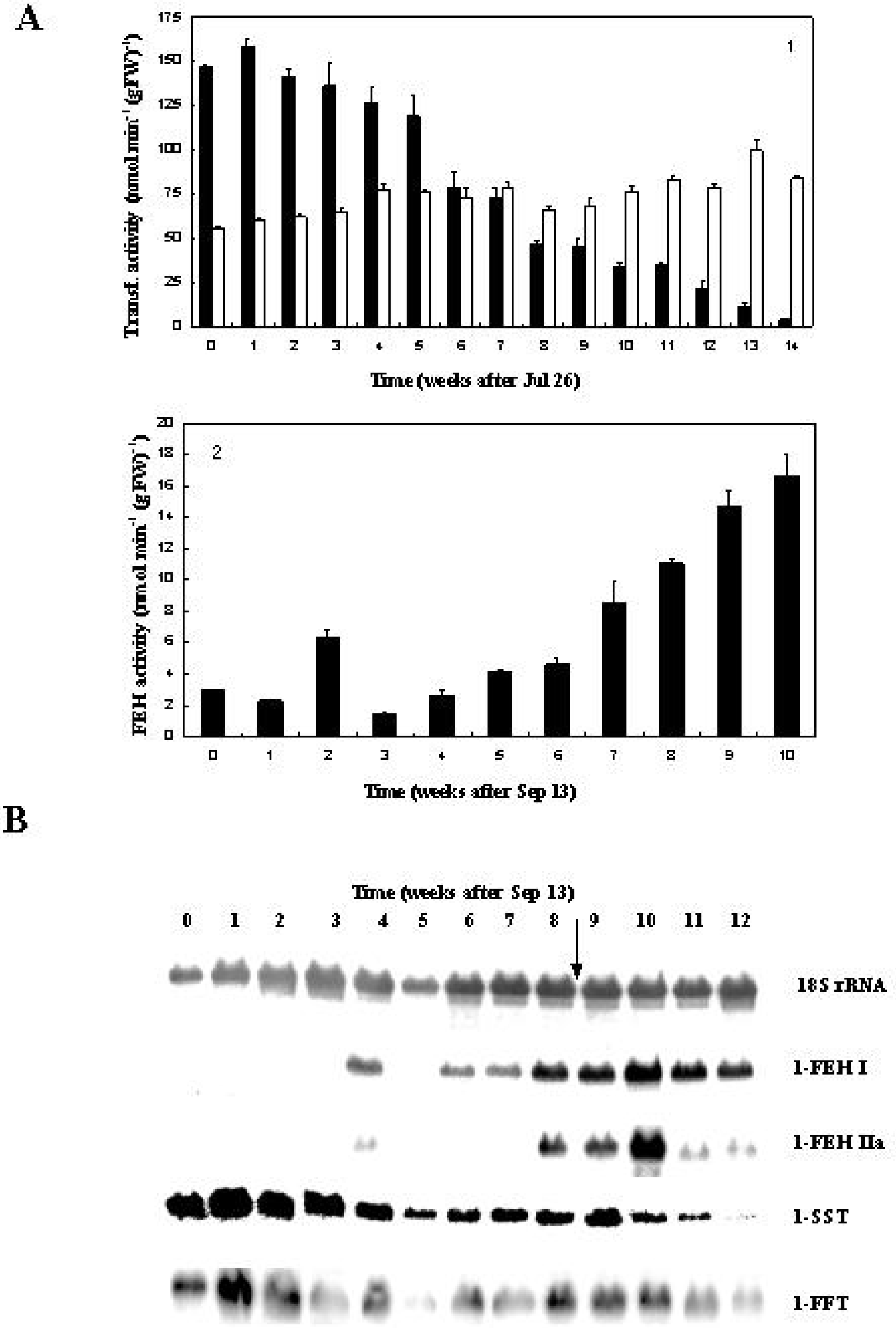

**FIGURE 3.** (A) The transferase activities (1) of 1-SST (black bars, 100 mM sucrose as substrate) and 1-FFT (white bars, 10 mM 1-kestose as substrate) in chicory roots during field growth. The hydrolase activities (2) of 1-FEH (1% chicory inulin as substrate) in chicory roots throughout autumn. Bars represent standard errors for n = 3. (B) Expression of chicory genes. Northern blot containing RNA from mature field-grown chicory roots throughout autumn and subsequent storage for 3 weeks in a cold room (arrow indicates start of storage). 1-SST, 1-FFT, 1-FEH I, 1-FEH IIa, and 18S rRNA specific probes were used.

proof of the involvement of sucrose in fructan biosynthetic gene induction comes from incubation of detached leaves in sucrose solutions[36,76], resulting in fructan biosynthesis. Sugars have important signaling functions throughout all stages of the plant's life cycle[77]. It was demonstrated that sugars (sucrose and, to a lesser extent, glucose and fructose) are able to induce fructan biosynthetic genes in grasses[78,79,80], but regulation likely involves multiple factors. It was suggested that the regulation of fructan synthesis in barley leaves occurs independently of hexokinase (no induction by mannose, for example) and is probably based on the sensing of sucrose. It was found that the structurally related disaccharide trehalose can replace sucrose as a regulatory compound[78]. It was further demonstrated that protein kinase and protein phosphatase inhibitors can inhibit the gene induction by sugars, indicating that protein kinase and protein phosphatase activities take part in the chain of events that intervene in the induction of fructan biosynthesis[81].

Defoliation also causes an increase in the activity of FEH in stubble of *L. perenne*. This FEH activity was found to be strongly inhibited by an inhibitor of the biosynthesis of gibberellin: uniconazole. The inhibition could be overcome by a subsequent treatment with gibberellic acids (GAs). These data support the view that GAs might play a role in the regulation of FEH activity[82].

### CONCLUSION AND PROSPECTIVES

Fructans widely occur in plants as a reserve carbohydrate. At least inulin biosynthesis and breakdown have now been unraveled. Fructan breakdown enzymes are probably derived from an ancient cell-wall invertase, while fructan biosynthetic enzymes evolved from a vacuolar-type invertase. All these fructan enzymes differ in their donor and acceptor substrate specificities. Site-directed mutagenesis will teach us how these enzymes function and which molecular determinants control their specificity. By genetical engineering, this will probably allow us to control fructan metabolism in chicory (antisense FEH) or, alternatively, to produce structurally different types of fructans in transgenic crops.

### ACKNOWLEDGMENTS

W. Van den Ende is a Postdoc supported by a grant from the Fund for Scientific Research (FSR, Flanders, no. G.0238.98).
